# Relation of climatic exposures with intrahepatic cholestasis of pregnancy in a two-center retrospective cohort

**DOI:** 10.3389/fendo.2026.1924211

**Published:** 2026-08-14

**Authors:** Xiao Chen, Chang Zou, Yang Liu, Ruru Zhao, Hanxiang Sun, Xiaosong Liu, Qinxin Shen, Yuanyuan Yang, Qiaoling Du

**Affiliations:** Shanghai Key Laboratory of Maternal Fetal Medicine, Shanghai Institute of Maternal-Fetal Medicine and Gynecologic Oncology, Shanghai First Maternity and Infant Hospital, School of Medicine, Tongji University, Shanghai, China

**Keywords:** air polluation, environmental exposure, intrahepatic cholestasis during pregnancy, retrospective cohort study, temperature

## Abstract

**Objective:**

To investigate relation between climatic exposures and intrahepatic cholestasis of pregnancy (ICP) in Chinese cities Shanghai and Sanya.

**Background:**

ICP is a pregnancy-specific liver disease associated with adverse fetal outcomes. Although the etiology of ICP remains unclear, climatic exposures are increasingly recognized as one of the critical correlates in ICP onset.

**Methods:**

This is a two-center retrospective cohort study included 48, 081 pregnant women from Shanghai (31°N, temperate) and Sanya (18°N, tropical). Baseline demographic data, pregnancy outcomes and meteorological data were collected. Generalized additive models (GAMs) were employed to analyze non-linear associations between environmental exposures and ICP risk, with statistical comparisons of baseline characteristics and complications between ICP and non-ICP groups.

**Results:**

GAM analysis revealed that temperature, sunlight and PM_2.5_ exhibited positive gestation-age-dependent non-linear correlations with ICP risk in Shanghai, while relative humidity showed an inverse correlation. No meteorological exposures showed significant associations with ICP in Sanya. Sunlight could amplify heat-related exposure signals alongside high temperature. In Shanghai, pregnancies conceived in February and March showed the highest ICP incidence, while Sanya showed no significant seasonal fluctuation and maintained a higher overall ICP incidence than Shanghai under constant warm climate. Thalassemia prevalence was higher in ICP cases compared to controls in both regions (Sanya: 31.8% vs. 17.9%; Shanghai:1.0% vs. 0.4%, all P<0.05). ICP patients in both cohorts demonstrated shortened gestational age, with Shanghai exhibiting a more pronounced gestational age reduction correlated with higher cesarean delivery rates.

**Conclusion:**

High temperature, intense sunlight exposure, and high PM_2.5_ concentration across preconception and mid-pregnancy serves as major climatic correlates of ICP incidence.

## 1 Introduction

Intrahepatic cholestasis of pregnancy (ICP) is a pregnancy-specific liver disease characterized by maternal pruritus typically occurring in the second or third trimester, accompanied by elevated serum total bile acid (TBA) levels with or without abnormal liver function tests (1). The global incidence of ICP varies widely in different regions, ranging from < 1% to 27.6% (2–6). Beyond causing significant maternal discomfort, ICP increases the risk of serious adverse fetal outcomes, including preterm birth, meconium-stained amniotic fluid, fetal distress, neonatal respiratory distress syndrome, and even fetal death (7, 8).

Although the etiology of ICP remains incompletely elucidated, emerging evidence increasingly indicates that climatic exposures may play roles in it (1, 9). Epidemiological studies have documented a distinct seasonal pattern in ICP incidence, with substantially higher diagnosis rates observed during winter months compared to summer (10, 11), suggest that ambient temperature, relative humidity, and daylight duration may be associated with ICP onset. Give the profound impacts of climatic conditions on maternal health outcomes, further investigation is warranted. Additionally, air pollution has been demonstrated to be associated with elevated ICP risk (12, 13).

Geographic latitude has also been identified as a significant correlate influencing liver disease incidence, as suggested by Prince et al., who found higher incidences of primary biliary cholangitis (PBC) and autoimmune hepatitis in northern regions of the United Kingdom (14), and French et al. documented a distinct latitudinal gradient in PBC incidence across Australia (15).

In our two-center retrospective analysis, we observed significantly different distribution of ICP incidence between Shanghai (31°N, investigated at Shanghai First Maternity and Infant Hospital) and Sanya (18°N, investigated at Sanya Maternal and Child Health Hospital), with Sanya demonstrating substantially higher ICP incidence than Shanghai. To explore how climatic exposures, clinical characteristics, and region-specific complications such as thalassemia contribute to the heterogeneous distribution of ICP incidence across these geographically distinct regions, we compared population characteristics between the two cohorts and established generalized additive model (GAM) incorporating ambient temperature, sunlight exposure, PM_2.5_ concentration, and relative humidity. Our analysis revealed that in Shanghai, the temperate region with significant annual temperature fluctuations, temperature, sunlight and PM_2.5_ showed positive time-dependent correlations with ICP risk, while relative humidity exhibited an inverse correlative pattern. Meanwhile, thalassemia, a complication with notably high regional prevalence in Hainan province, constitutes a major correlate for ICP diagnosis in Sanya. Sunlight exposure demonstrated no significant association with ICP development in either region.

These findings on region-specific correlates of ICP onset provide clinical guidance for preconception counseling and gestational risk-tailored management across geographically distinct populations. The results underscore the critical importance of pre-pregnancy screening for hereditary disease susceptibility and validate the clinical necessity of baseline liver function assessment for high-risk populations, thereby enhancing the overall efficiency of clinical prevention and intervention strategies for ICP. Given the accelerating pace of climate change and its potential impacts on maternal health, investigating the relationship between climatic exposures and ICP holds significant public health importance.

## 2 Methods

### 2.1 Study design and participants

This retrospective cohort study utilized data from two geographically distinct locations in China: 1) Sanya Maternal and Child Health Hospital (low-latitude tropical city, 18.217°N, 109.5°E); 2) Shanghai First Maternity and Infant Hospital (high-latitude temperate city, 31.143°N, 121.805°E), conducted in accordance with the Declaration of Helsinki. The study involving human participants received institutional approval from the Institutional Review Boards of Shanghai First Maternity and Infant Hospital (approval numbers: KS25405) and Hainan Medical University (approval numbers: 731). Due to the retrospective design of the study, written informed consent was waived, and all measures were implemented to ensure participant privacy and maintain anonymity.

Eligible participants were pregnant women who conceived between 1 April 2018 and 30 September 2022 with conception date estimated using the first day of the last menstrual period (LMP). All included participants received prenatal care and delivered at one of the two study hospitals. To minimize potential selection bias, the following exclusion criteria were systematically applied:

- Residence in the local area for less than one year.
- Twin or multiple gestation.
- History of chronic hepatitis or hepatobiliary stone disease.
- Gestational age at delivery less than 28 weeks.
- Lack of detailed residential address or incomplete medical records.

**Figure 1:**
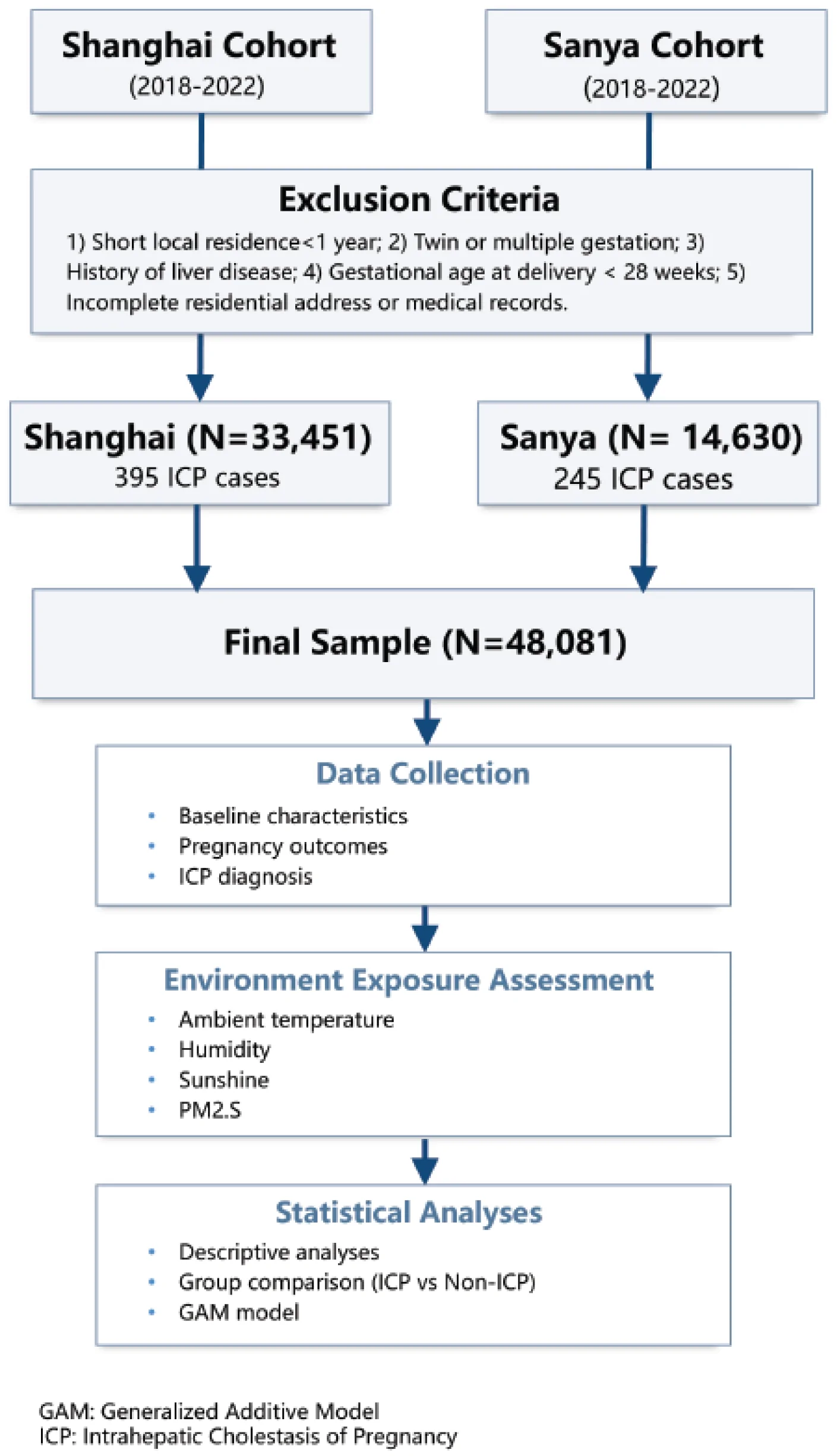
Flowchart of study design and participant selection. This two-center retrospective study included pregnant women from Shanghai Cohort and Sanya Cohort (2018–2022). ICP: intrahepatic cholestasis of pregnancy; GAM: generalized additive model.

After exclusion, there were 33, 451 eligible singleton pregnancy cases enrolled from Shanghai First Maternity and Infant Hospital, and 14, 630 from Sanya Maternal and Child Health Hospital (**Figure 1**).

### 2.2 Environmental data and exposure assessment

All meteorological variables were collected and processed at a daily temporal resolution. At the spatial scale, key climatic parameters, including peak sunshine hours, mean temperature, and relative humidity were obtained from China National Meteorological Information Center (https://data.cma.cn/) based on measurements recorded at local meteorological monitoring stations in Shanghai and Sanya (**Supplementary Figures S1**). To estimate the spatial distribution of PM2.5 concentrations, data from both the China National Meteorological Information Center and the Wheat A platform (https://wheata.cn/) were integrated, and spatial interpolation was performed using the Wheat A tool to generate raster datasets at a spatial resolution of 0.75° × 0.75°. Daily meteorological records were spatially linked to each participant according to residential geocoordinates via a nearest-neighbor assignment approach.

To characterize the time-varying environmental exposures across the preconception to mid-gestational periods, we defined eight consecutive exposure windows (*t*) with equal duration. Mean meteorological levels over each complete window were calculated to capture sustained ambient exposure. The predefined windows were specified as follows:

- *t* = -1: 28 days preceding the first day of the LMP.
- *t* = 1: LMP to gestational day 27.
- *t* = 2: gestational days 28–55.
- *t* = 3: gestational days 56–83.
- *t* = 4: gestational days 84–111.
- *t* = 5: gestational days 112–139.
- *t* = 6: gestational days 140–167.
- *t* = 7: gestational days 168–195.

Daily exposure levels for temperature, relative humidity, peak sunshine hours, and PM_2.5_ were assigned to each participant based on residential location.

### 2.3 Data collection

Data were collected from both prenatal interviews and electronic medical records at both Sanya Maternal and Child Health Hospital and Shanghai First Maternity and Infant Hospital. At prenatal registration, all participants were interviewed in person to obtain baseline sociodemographic and clinical information, including maternal age, occupation, personal and family medical history, as well as detailed obstetric history (e.g., gravidity, parity). Following delivery, additional information regarding pregnancy outcomes and complications were systematically extracted from the hospital electronic medical record systems. These data included LMP, gestational age (confirmed by ultrasound examination), pregnancy-related complications (e.g., gestational diabetes and hypertensive disorders), mode of delivery. Primary outcome of this study was diagnosis of ICP, according to *Guidelines for clinical diagnosis, treatment and management of intrahepatic cholestasis of pregnancy (2024)* (16) as a Chinese standard guideline, defined as an elevated fasting serum total bile acid (TBA) concentration (exceeding 10 μmol/L) accompanied by pruritus.

### 2.4 Statistical methods

Categorical variables were presented as frequencies and proportions with between-group differences assessed using the Chi-square test. Continuous variables following normal distribution are presented as mean ± standard deviation, whereas non-normally distributed continuous variables are presented as median [interquartile range (IQR)]. Inter-group differences for continuous variables were evaluated using Student’s t-test or the Mann-Whitney U test, as appropriate. The distribution characteristics of exposures at different time points were described; one-way ANOVA or the Chi-square test was used to compare differences in exposures across time periods. GAMs with binomial logit link were constructed to assess the non-linear associations between meteorological exposures and the risk of ICP. Two key confounders were retained for adjustment in all final GAMs: maternal age at conception (continuous smooth term) and thalassemia status (binary categorical term, chosen based on the former logistic regression). Cubic regression splines with (k=7) were adopted for meteorological exposures, with smooth terms of gestational age *t* fitted separately for each climate variable via the *by* argument to capture window-varying exposure effects. All models were fitted with Restricted Maximum Likelihood (REML) estimation.

All statistical analyses were conducted using R version 4.4.1. A P-value less than 0.05 was considered statistically significant.

## 3 Results

### 3.1 Population characteristics comparison between Shanghai and Sanya cohorts by ICP status

This study included a total of 48, 081 eligible pregnant women, comprising 33, 451 from Shanghai First Maternity and Infant Hospital and 14, 630 from Sanya Maternal and Child Health Hospital (**Figure 1**). The overall ICP incidence rate combined the two centers was approximately 1.33%, with 640 cases were diagnosed with ICP (**Table 1**).

**Table 1:** Sociodemographic and obstetric characteristics of all participants stratified by ICP status in the overall combined cohort.

Characteristics	All pregnant (n = 48, 081)	ICP (n = 640)	Non-ICP (n = 47, 441)	P value (ICP vs non-ICP)
Maternal age (years)	30.3 ± 4.1	30.1 ± 4.3	30.3 ± 4.1	0.220
Gestational age (weeks)	39.0 ± 1.4	38.0 ± 1.7	39.0 ± 1.4	<0.001
Occupation, n (%)				
-Other	17, 228 (35.8%)	238 (37.2%)	16, 990 (35.8%)	0.002
-Unemployed	7, 596 (15.8%)	131 (20.5%)	7, 465 (15.7%)	
-Intellectual labor	20, 088 (41.8%)	240 (37.5%)	19, 848 (41.8%)	
-Manual labor	3, 169 (6.6%)	31 (4.8%)	3, 138 (6.6%)	
History of reproduction	1 (1, 2)	1 (1, 2)	1 (1, 2)	0.690
Parity	0 (0, 1)	0 (0, 1)	0 (0, 1)	0.792
Number of previous abortions	0 (0, 1)	0 (0, 1)	0 (0, 1)	0.824

Sociodemographic and obstetric characteristics of all study participants by intrahepatic cholestasis of pregnancy (ICP) status (n = 48, 081). Data are presented as mean ± standard deviation for continuous variables (maternal age, gestational age) and median (interquartile range) for reproductive history variables. Categorical variables (occupation) are shown as n (%). P-values derived from independent t-tests, Mann-Whitney U tests, or chi-square tests as appropriate for comparisons between ICP (n = 640) and non-ICP (n = 47, 441) groups. ICP: intrahepatic cholestasis of pregnancy.

In terms of demographic characteristics, maternal age at conception was comparable between the ICP and non-ICP groups (30.1 ± 4.3 years vs. 30.3 ± 4.1 years, P = 0.220). Occupational distribution differed significantly between groups (P = 0.002). Gestational age at delivery was significantly lower in the ICP group (38.0 ± 1.7 weeks vs. 39.0 ± 1.4 weeks, P<0.001). Reproductive history including gravidity, parity, and previous abortion times showed no significant differences between groups (P = 0.690, 0.792, and 0.824, respectively), indicating no significant association between these obstetric factors and ICP occurrence.

Site-specific differences in baseline characteristics were observed between Sanya and Shanghai cohorts (**Table 2**). ICP incidence showed different distribution (Sanya 1.67% and Shanghai 1.18%). Reduced gestational age at delivery was a consistent feature of ICP in both cohorts. The reduction was 0.7 weeks in Sanya cohort (38.2 ± 1.7 weeks vs. 38.9 ± 1.3 weeks, P<0.001) and 1.2 weeks in the Shanghai cohort (37.9 ± 1.7 vs. 39.1 ± 1.4 weeks, P<0.001), with the larger reduction in Shanghai primarily attributable to more active obstetric intervention through cesarean delivery. Higher unemployment in the ICP group was observed only in the Sanya cohort (44.5% vs. 36.8%, P = 0.009), with no such occupational disparity evident in Shanghai.

**Table 2:** Sociodemographic, obstetric characteristics, and adverse outcomes stratified by ICP status in Sanya and Shanghai sub-cohorts.

Sanya cohort	ICP (n = 245)	Non-ICP (n = 14, 385)	P value
Maternal age (years)	29.7 ± 5.1	29.3 ± 4.8	0.215
Gestational age (weeks)	38.2 ± 1.7	38.9 ± 1.3	<0.001
Occupation, n (%)				
-Other	93 (38.0)	5, 283 (36.7)	0.009
-Unemployed	109 (44.5)	5, 291 (36.8)	
-Intellectual labor	43 (17.6)	3, 757 (26.1)	
-Manual labor	0 (0.0)	54 (0.4)	
History of reproduction	2 (1, 3)	2 (1, 3)	0.824
Parity	0 (0, 1)	0 (0, 1)	0.931
Number of previous abortions	0 (0, 1)	0 (0, 1)	0.738
Thalassemia, n (%)	78 (31.8)	2, 580 (17.9)	<0.001
Diabetes, n (%)	60 (24.5)	3, 263 (22.7)	0.503
Gestational hypertension, n (%)	22 (9.0)	631 (4.4)	<0.001
Preterm birth, n (%)	36 (14.7)	679 (4.7)	<0.001
Cesarean section, n (%)	110 (44.9)	5, 914 (41.1)	0.233

Shanghai cohort	ICP (n = 395)	Non-ICP (n = 33, 056)	P value
Maternal age (years)	30.4 ± 3.7	30.8 ± 3.8	0.032
Gestational age (weeks)	37.9 ± 1.7	39.1 ± 1.4	<0.001
Occupation, n (%)			
-Other	145 (36.7)	11, 707 (35.4)	0.610
-Unemployed	22 (5.6)	2, 174 (6.6)
-Intellectual labor	197 (49.9)	16, 091 (48.7)
-Manual labor	31 (7.8)	3, 084 (9.3)
History of reproduction	1 (1, 2)	1 (1, 2)	0.016
Parity	0 (0, 0)	0 (0, 0)	0.170
Number of previous abortions	0 (0, 0)	0 (0, 0)	0.072
Thalassemia, n (%)	4 (1.0)	116 (0.4)	0.029
Diabetes, n (%)	43 (10.9)	4, 156 (12.6)	0.315
Gestational hypertension, n (%)	41 (10.4)	1, 947 (5.9)	<0.001
Preterm birth, n (%)	66 (16.7)	1, 523 (4.6)	<0.001
Cesarean section, n (%)	263 (66.6)	14, 943 (45.2)	<0.001

Baseline characteristics of the Sanya and Shanghai cohort subsets stratified by ICP status. Continuous variables expressed as mean ± SD; categorical variables as n (%). Statistical comparisons between ICP and non-ICP groups performed using appropriate parametric or non-parametric tests. ICP: intrahepatic cholestasis of pregnancy.

### 3.2 Comorbidities and pregnancy outcomes in Sanya and Shanghai ICP cohorts

**Figure 2:**
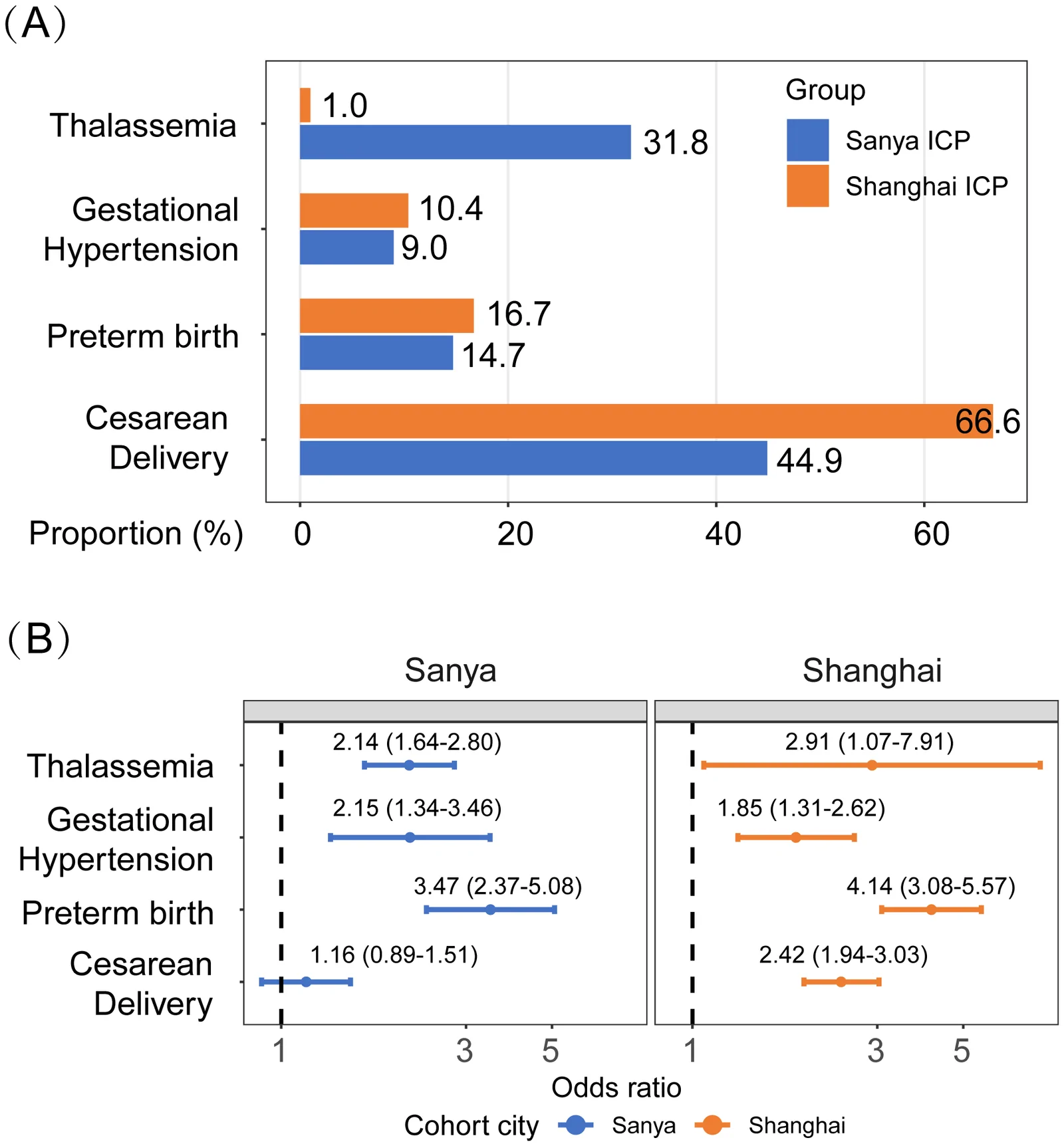
Comparison of comorbidities and adverse outcomes between ICP patients in Sanya and Shanghai. **(A)** Bar chart comparing the prevalence of comorbidities and adverse outcomes between ICP patients from Sanya and Shanghai. Blue bars represent Sanya ICP cases, orange bars represent Shanghai ICP cases; percentage values are labelled above each bar. **(B)** Forest plot of odds ratios (OR) for clinical complications and adverse outcomes comparing ICP versus non-ICP women in the two cohorts. Horizontal bars indicate 95% confidence intervals, and the vertical dashed line at OR = 1 denotes no statistically significant difference. Marked regional heterogeneity was observed in thalassemia prevalence and cesarean delivery risk differences.

The prevalence of comorbidities and adverse pregnancy outcomes exhibited both consistent trends and prominent site-specific differences between the Sanya and Shanghai cohorts (**Table 2**, **Figure 2**). For thalassemia, a substantially higher proportion was observed in ICP cases relative to controls in both regions. The elevation was particularly pronounced in the Sanya cohort (31.8% vs. 17.9%, P<0.001), whereas the difference remained statistically significant but with considerably lower overall prevalence in Shanghai (1.0% vs. 0.4%, P = 0.029). Regarding hypertensive disorders in pregnancy, ICP women in both cohorts demonstrated significantly higher prevalence compared to corresponding controls (Sanya: 9.0% vs. 4.4%, P<0.001; Shanghai: 10.4% vs. 5.9%, p<0.001), while the rate of gestational diabetes showed no significant difference between ICP and control groups in either Sanya (24.5% vs. 22.7%, P = 0.503) or Shanghai (10.9% vs. 12.6%, P = 0.315) (**Figure 2**).

In terms of pregnancy outcomes, preterm birth rates were significantly elevated among ICP patients in both cohorts (Sanya: 14.7% vs. 4.7%, p<0.001; Shanghai: 16.7% vs. 4.6%, p<0.001). Cesarean delivery rates exhibited distinct regional patterns: no significant difference was observed between ICP and control women in Sanya (44.9% vs. 41.1%, P = 0.233), whereas Shanghai’s ICP group showed a substantially higher cesarean delivery rates compared to controls (66.6% vs. 45.2%, P<0.001), reflecting notably more active obstetric intervention strategies for local ICP pregnancies (**Figure 2**).

### 3.3 Site-specific differences in non-linear relationships between environmental exposures and ICP risk

**Figure 3:**
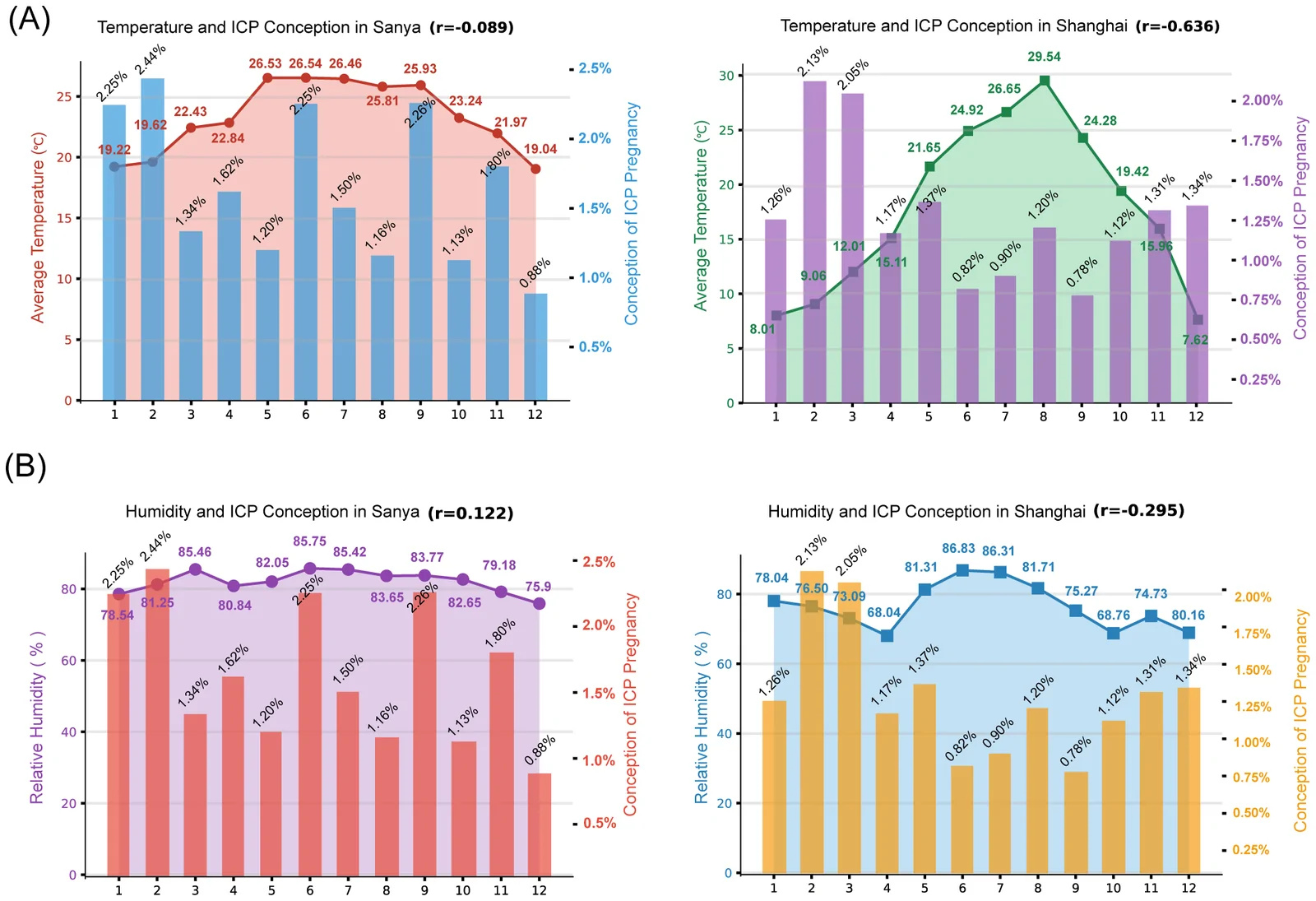
Monthly seasonal variation and the time distribution of ICP pregnancy conception in Sanya and Shanghai (2020). **(A)** Monthly average temperature and ICP conception rates in Sanya (left) and Shanghai (right). **(B)** Monthly average relative humidity and ICP conception rates in Sanya (left) and Shanghai (right). The bar charts represent the monthly conception rates of ICP pregnancy, and the line charts indicate the corresponding meteorological variables. The x-axis represents the LMP month. Pearson correlation coefficients (r) are shown for each city in the subtitles. ICP, intrahepatic cholestasis of pregnancy.

**Figure 3** illustrates the monthly ICP incidence by conception rates by LMP in Sanya and Shanghai, in the year of 2020. The results show that the most common conception time of ICP pregnancy in Shanghai is in February to March (2.13%, 2.05%), whereas the lowest incidence is observed in LMP from June to September (the lowest 0.78% in September). Meanwhile, Sanya features a stable, consistently warm tropical climate, and displayed a persistently higher overall ICP incidence than Shanghai, with no obvious temporal fluctuation across calendar months.

**Figure 4:**
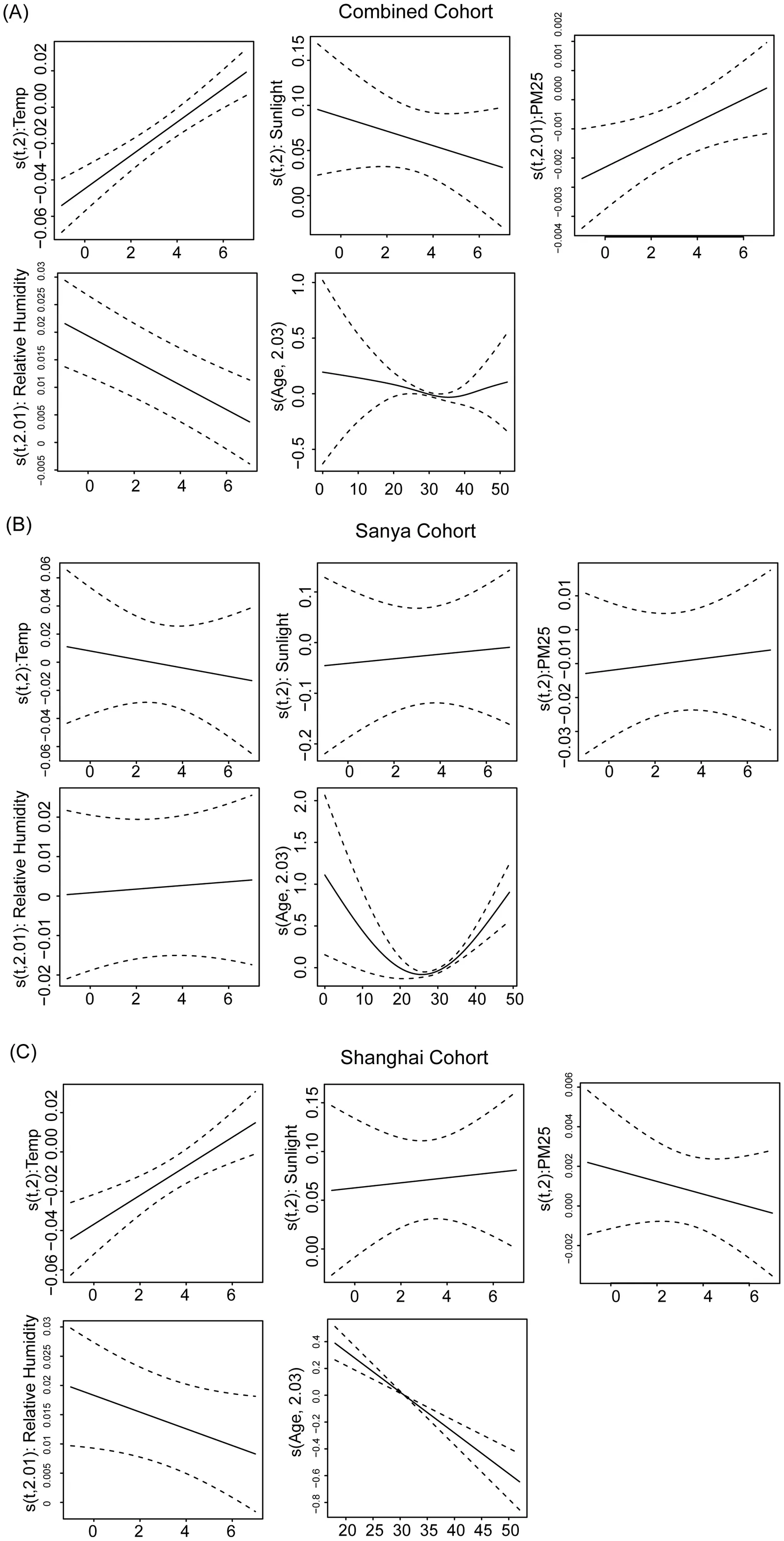
GAM smooth curves for the non-linear associations between environmental exposures, maternal age, and ICP risk. GAM smooth curves showing non-linear associations of sunlight, PM_2.5_, temperature, relative humidity and maternal conception age with ICP risk in the combined Shanghai-Sanya cohort **(A)**, and Sanya cohort **(B)**, Shanghai cohort **(C)**, respectively. Y-axis: GAM smooth term estimate; edf values in parentheses. ICP: intrahepatic cholestasis of pregnancy; edf: effective degrees of freedom.

GAMs revealed distinct patterns of non-linear associations between environmental exposures and ICP risk, with marked heterogeneity across cohorts (**Table 3**, **Figure 4**). In the combined Shanghai-Sanya dataset, significant and gestation-age-dependent non-linear associations were identified for temperature (edf = 2.003, P<0.001), PM_2.5_ (edf = 2.015, P = 0.001), and relative humidity (edf = 2.003, P<0.001) (**Table 3**, **Figure 4A**). All four environmental exposures showed effective degrees of freedom near 2, indicative of simple single-inflection nonlinear curves rather than complex oscillating trends. The risk contributions of ambient temperature were weakest during the 28-day preconception window (t=-1) and rose monotonically across sequential gestational windows, with the strongest effects observed in early-to-mid pregnancy. Risk related to sunlight exposure followed a U-shaped trajectory, preconception sunlight conferred elevated ICP risk, risk declined modestly in early gestation, then rebounded sharply to higher levels in mid-to-late pregnancy. PM_2.5_ exerted progressively stronger enhancing effects as gestation advanced, with incremental increases from preconception through 27^+6^ gestational week. Relative humidity displayed a steady downward trend across all exposure windows, presenting its protective effect against ICP strengthened continuously with advancing pregnancy. Conception age showed no significant non-linear association with ICP risk in the pooled analysis.

**Table 3:** Non-linear effects of environmental exposures and maternal characteristics on ICP risk estimated by GAM.

Smooth term	edf	Ref.df	Chi-sq	p-value
Combined Cohort of Shanghai and Sanya				
Temperature (°C)	2.003	2.006	53.795	0.000^***^
Sunlight (h)	2.005	2.010	13.773	0.001^**^
PM_2.5_(µg/m³)	2.015	2.029	10.306	0.006^**^
Relative Humidity (%)	2.007	2.013	33.385	0.000^***^
Conception Age (years)	2.033	2.543	5.028	0.165
Sanya Cohort				
Temperature (°C)	2.000	2.001	0.296	0.863
Sunlight (h)	2.000	2.001	0.368	0.832
PM_2.5_(µg/m³)	2.000	2.001	1.837	0.399
Relative Humidity (%)	2.000	2.001	0.153	0.927
Conception Age (years)	1.927	1.995	32.016	0.000^***^
Shanghai Cohort				
Temperature (°C)	2.002	2.004	23.429	0.000^***^
Sunlight (h)	2.002	2.004	12.430	0.002^**^
PM_2.5_(µg/m³)	2.002	2.004	1.599	0.450
Relative Humidity (%)	2.002	2.004	16.784	0.000^***^
Conception Age (years)	1.003	1.007	39.155	0.000^***^

Non-linear effects of environmental exposures (temperature, sunlight, PM_2.5_, relative humidity) and maternal characteristics (conception age) on the risk of ICP, estimated using generalized additive models (GAM). The table reports the effective degrees of freedom (edf), reference degrees of freedom (Ref.df), chi-square statistics, and p-values for each smooth term. Results are presented for the combined cohort (Shanghai and Sanya), and for the individual Sanya and Shanghai cohorts. Higher edf indicates greater non-linearity. Significant terms are indicated by *** (p < 0.001) or ** (p < 0.01).

Stratified cohort analyses revealed pronounced site-specific disparities. In the Sanya cohort, conception age was the only significant correlate (edf = 1.927, P<0.001), while all four environmental exposures demonstrated no significant association. Smooth curves for temperature, sunlight, PM_2.5_, and relative humidity remained fluctuating slightly around 0 during the whole pregnancy period, with no obvious time-dependent trend (**Table 3**, **Figure 4B**). Conversely, the Shanghai cohort demonstrated strong non-linear associations for both temperature (edf = 2.002, P<0.001), sunlight duration (edf = 2.002, P = 0.002), and relative humidity (edf = 2.002, P<0.001) with ICP risk (**Table 3**, **Figure 4C**). Consistent with the former combined analysis, associations of temperature with ICP risk intensified gradually from preconception through early-to-mid gestation, and relative humidity’s protective effect strengthened steadily with advancing pregnancy. Sunlight exhibited a comparable U-shaped profile to the combined sample cohort, with elevated risk in preconception and mid-gestation, and mild protection in early pregnancy. PM_2.5_ showed no independent association with ICP risk in Shanghai (P = 0.450).

## 4 Discussion

This two-center retrospective cohort study systematically evaluated associations between climatic environmental exposures and the risk of ICP, revealing significant regional heterogeneity. Specifically, in temperate Shanghai with prominent seasonal temperature fluctuations, ambient temperature, sunlight and PM_2.5_ exhibited positive time-dependent non-linear correlations with ICP risk, while relative humidity showed an inverse correlation, whereas tropical city of Sanya with consistently warm conditions, climatic factors demonstrated no significant associations with ICP. Instead, thalassemia emerged as the predominant correlate in this region. These findings emphasize the critical importance of geographic specificity in understanding ICP etiology and developing region-tailored prevention strategies.

A key finding of this study is the strong association of heat during pregnancy periods with ICP incidence in Shanghai. Heat exposure may exacerbate oxidative stress and disrupt bile acid homeostasis, facilitating progressive bile acid accumulation. By stratifying ICP incidence by conception month, we found that pregnancies starting in February and March in Shanghai carried the highest ICP risk, corresponding to intensive heat exposure during subsequent summer months, whereas conceptions occurring from June to September showed lower incidence, with gestational periods covering cooler seasons. By analyzing ICP incidence according to LMP month, we found that in Shanghai, the highest ICP conception rate occurred in February and March, corresponding to the strongest heat exposure in summer time from May to August during pregnancy, whereas the lowest incidence was observed in LMP June to September, when the pregnancy period fell in cooler months. This seasonal pattern aligns closely with the GAM results, which demonstrated significant non-linear associations between temperature, relative humidity, and ICP risk in Shanghai. Sunlight showed a weak time-dependent non-linear correlation in Shanghai. Rather than acting independently, intense sunlight reinforces ambient heat load and amplifies heat-related stress. Relative humidity exhibited an inverse correlation with ICP risk. Higher humidity may buffer abrupt temperature fluctuations and mitigate extreme heat stress, thereby attenuating the adverse impacts of temperature fluctuation on hepatic metabolism. Although ICP has been traditionally reported with higher diagnostic frequency during winter months (10, 11), this likely reflects the timing of clinical detection rather than true disease pathogenesis. ICP doesn’t emerge abruptly in late pregnancy, instead, bile acid dysregulation may develop progressively throughout gestation (17). In Shanghai, pregnancies culminating in winter inevitably involve sustained exposure to warmer and more humid conditions during gestation, resulting in prolonged heat exposure. This pattern aligns with our findings and may also explain the higher ICP incidence observed in tropical regions such as Sanya. Potential biological mechanisms underlying these associations include disrupted bile acid metabolism, compositional alterations in gut microbiota, and changes in metabolic hormone secretion (e.g., GLP-1) (18–20).

In contrast, no significant associations between environmental exposures and ICP risk were observed in the Sanya cohort. This may be explained by the tropical maritime climate (21), defined by persistently elevated temperature and humidity with minimal seasonal variation, which substantially reduces exposure variability and limits its discriminatory power for disease risk. In both regions, thalassemia prevalence was significantly elevated in ICP patients, particularly in Sanya, which demonstrates notably high regional thalassemia incidence (22). The underlying mechanism may involve chronic hemolysis and iron overload characteristic of thalassemia (23–25), which increases hepatic metabolic burden and impairs bile acid metabolism, thereby elevating ICP risk. These findings carry substantial clinical implications regarding genetic susceptibility in ICP pathogenesis.

PM_2.5_ exhibited positive time-dependent non-linear correlations with ICP risk in the combined dataset, yet no comparable associations emerged in separate regional analyses. Experimental evidence suggests PM_2.5_ may trigger hepatic low-grade inflammation and oxidative stress, interfering with bile acid excretion (26). The discrepancy between the combined and single-center results is likely driven by heterogeneous PM_2.5_ background concentrations across the two geographically distant cities, generating sufficient inter-site variation to capture a weak overall correlation. Within each city, however, limited temporal fluctuations in PM_2.5_ weakened the ability to identify independent associations after adjustment for co-occurring meteorological factors.

Shortened gestational age at delivery was consistently observed across ICP patients in both cohorts. The gestational age reduction observed in the Sanya cohort appeared spontaneous in nature, whereas the more pronounced gestational age reduction in Shanghai likely reflects more proactive obstetric management strategies, specifically earlier pregnancy termination to mitigate fetal exposure to elevated maternal TBA concentrations, thereby reducing adverse fetal risks associated with ICP (27). It highlights potential disparities in obstetric management strategies and resource allocation across regions. In areas with more limited medical resources or less standardized monitoring protocols, delayed intervention may contribute to worse perinatal outcomes. Therefore, improving access to early screening, bile acid monitoring, and timely delivery decision-making should be emphasized, particularly in resource-constrained settings.

The contrasting findings between the two regions highlights the multifactorial nature of ICP etiology. In temperate regions such as Shanghai, environmental interventions (e.g., optimizing indoor temperature and relative humidity, reducing exposure to extreme climatic conditions that are sharply distinct from common environment) variations may contribute to ICP prevention. In tropical regions such as Sanya, enhanced emphasis should be placed on maternal risk assessment and genetic screening, particularly targeting thalassemia. This region-specific risk stratification provides a foundation for developing precision prevention strategies tailored to ICP pathogenesis in distinct geographic contexts.

This study has certain limitations. First, the retrospective design limits causal inference capabilities. Second, spatiotemporal exposure assignment was performed based on static residential geocoordinates using a nearest-neighbor matching strategy, without imposing a maximum distance cutoff between residences and meteorological stations. Comparable unconstrained matching approaches have been utilized in previous environmental epidemiological investigations (28). Nevertheless, only a small number of monitoring stations were accessible (3 stations in Shanghai and 2 stations covering the Sanya region), which may allocate analogous exposure estimates to participants living in the same city and limit spatial differentiation of exposure. Third, the residential addresses cannot fully capture mobility such as cross-district commuting, which may introduce exposure misclassification. Fourth, residual confounding from unmeasured factors including dietary habits and indoor environmental conditions cannot be entirely excluded. Future prospective studies incorporating individualized exposure assessment methodologies are warranted to address these limitations.

In conclusion, this study provides the first cross-latitudinal comparison of climatic and clinical correlates for ICP in China. The results demonstrate that combined high temperature and intense sunlight exposure across preconception and mid-gestational periods acts as a major climatic correlate of ICP risk in temperate regions, while higher relative humidity exerts an inverse correlative pattern by buffering extreme temperature fluctuation. These observations enrich the etiological understanding of ICP and provide evidence to support region-tailored ICP prevention and management strategies.

## 5 Conclusion

This cross-latitude cohort study confirms significant regional heterogeneity in the associations between environmental and individual factors and ICP risk across Shanghai and Sanya. In Shanghai, the city with significant temperature fluctuations compared to Sanya, ambient temperature, sunlight and PM_2.5_ show positive time-dependent correlations with ICP risk that intensify with advancing gestation, whereas relative humidity bears an inverse correlation with protective correlative effect strengthening across mid-pregnancy. No environmental correlates of ICP were observed in climatically stable Sanya. Shorter gestational length and higher gestational hypertension prevalence are shared clinical features of ICP pregnancies in both cohorts, and the more pronounced gestational shortening in Shanghai correlates with higher cesarean delivery rates. These findings advocate region-tailored ICP prevention, that subtropical areas with large temperature fluctuations should prioritize screening for women exposed to high temperature, intense sunlight and elevated PM_2.5_ in early- to mid-pregnancy.

## Data Availability

The raw data supporting the conclusions of this article will be made available by the authors, without undue reservation.
