# Supplementary material for: Relation of climatic exposures with intrahepatic cholestasis of pregnancy in a two-center retrospective cohort
Source: Front Endocrinol (Lausanne). 2026 Aug 14;17:1924211. doi: 10.3389/fendo.2026.1924211 (PMC13521805; doi:10.3389/fendo.2026.1924211)
Supplement: Supplementary file 1 [file Image1.pdf]

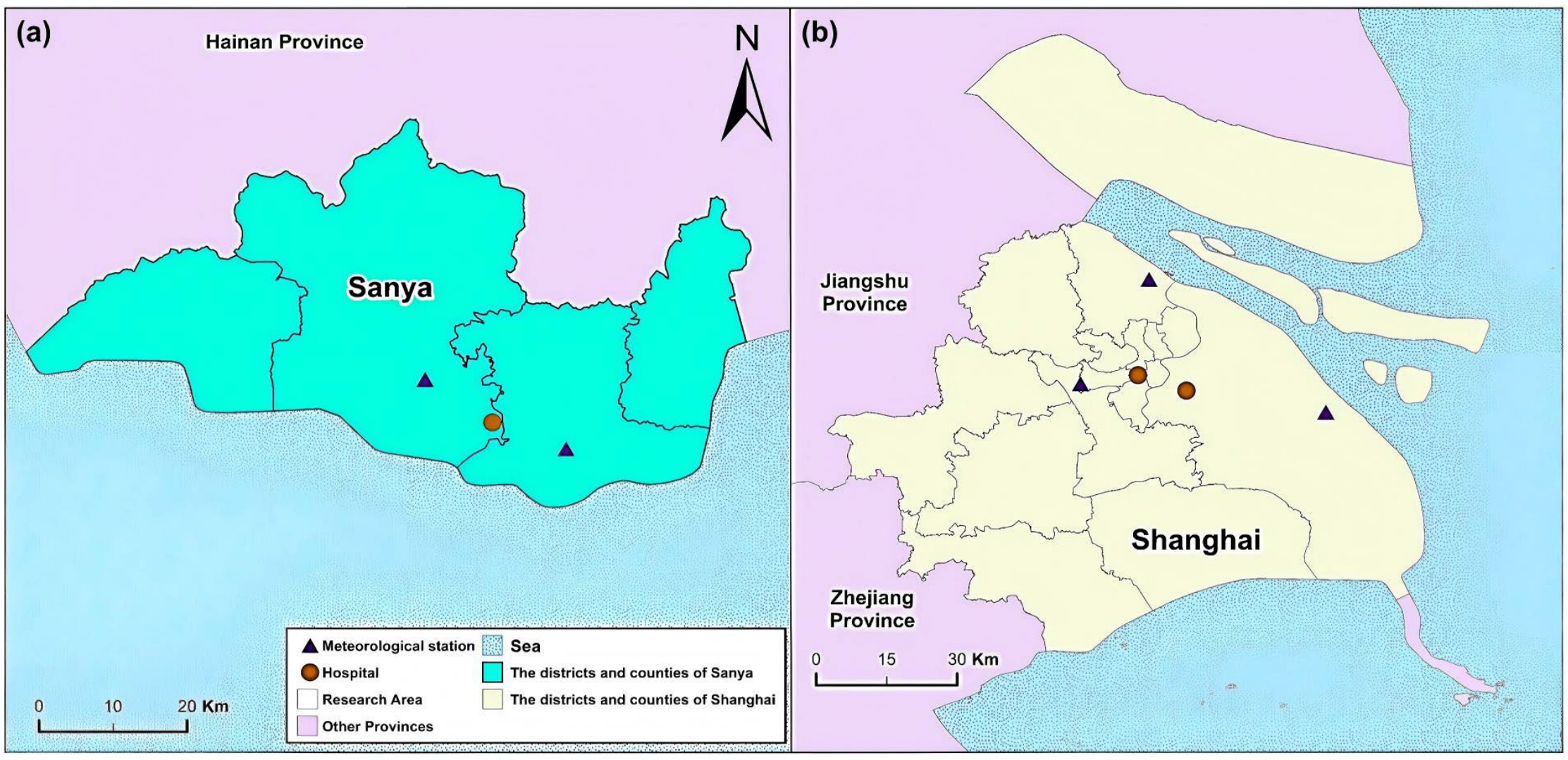

Fig S1. Spatial distribution of meteorological monitoring stations in Sanya and Shanghai

Geographical locations of collaborating hospitals and meteorological monitoring stations in Sanya and Shanghai, China. Spots denote hospital locations; triangle markers denote meteorological monitoring stations. The black scale bar represents actual distance on the map. Collaborating hospitals include the Sanya Maternal and Child Health Hospital in Sanya, and Jingan and Pudong campuses of Shanghai First Maternity and Infant Hospital in Shanghai.
